# Supplementary material for: TBX3 and EFNA4 Variant in a Family with Ulnar-Mammary Syndrome and Sagittal Craniosynostosis
Source: Genes (Basel). 2022 Sep 14;13(9):1649. doi: 10.3390/genes13091649 (PMC9498434; doi:10.3390/genes13091649)
Supplement: Supplementary file 1 [file genes-13-01649-s001.zip › Supplementary Table S1.pdf]

Table S1: 31 genes which were targeted for next generation sequencing in the proband.

Gene	OMIM ID
ALPL	171760
ALX4	605420
ASXL1	612990
CDC45	603465
CYP26B1	605207
EFNB1	300035
ERF	611888
FGFR1	136350
FGFR2	176943
FGFR3	134934
GLI3	165240
IFT122	606045
IFT43	614068
IL11RA	600939
MASP1	600521
MEGF8	604267
MSX2	123101
P4HB	176790
POR	124015
RAB23	606144
RECQL4	603780
SEC24D	607186
SKI	164780
TBX3	601621
TCF12	600480
TGFBR1	190181
TGFBR2	190182
TMCO1	213980
TWIST1	601622
WDR35	613602
ZIC1	600470
