# Supplementary material for: TBX3 and EFNA4 Variant in a Family with Ulnar-Mammary Syndrome and Sagittal Craniosynostosis
Source: Genes (Basel). 2022 Sep 14;13(9):1649. doi: 10.3390/genes13091649 (PMC9498434; doi:10.3390/genes13091649)
Supplement: Supplementary file 1 [file genes-13-01649-s001.zip › Supplementary Table S2_SpliceAI Prediction table.pdf]

	Where?	Score	Interpretation - up vs downstream interpretations flipped from the spliceai notes to account for the antisense coding strand.
Acceptor Loss	+147 bp	0.63	Hi confidence that the splice site 147 bp in the 5' direction will no longer function as an acceptor (63% increase in the chance that the position of coordinates plus 147 is no longer functioning as an acceptor [12:115,117,456])
Donor Loss	+1 bp	0.99	Hi confidence (Near certainty) that the splice site 1 bp in the 5' direction of the variant site will no longer function as a donor. (99% chance of loss of function as a donor site at variant coordinates plus 1 bp [12:115,117,310])
Acceptor Gain	(-)55 bp	0.02	There is no predicted likely acceptor site gained in a 500 bp window around the variant.
Donor Gain	(-)186 bp	0.32	Medium confidence that a site 186 bp in the 3' direction of the variant will now become the preferred donor site for the splice [12:115,117,123].
