# Supplementary material for: TBX3 and EFNA4 Variant in a Family with Ulnar-Mammary Syndrome and Sagittal Craniosynostosis
Source: Genes (Basel). 2022 Sep 14;13(9):1649. doi: 10.3390/genes13091649 (PMC9498434; doi:10.3390/genes13091649)
Supplement: Supplementary file 1 [file genes-13-01649-s001.zip › Supplementary Table S3_WGS_craniosynostosis gene list.pdf]

Gene	OMIM ID
ALPL	171760
ALX4	605420
ASXL1	612990
B3GAT3	606374
CD96	606037
CDC45	603465
CDT1	605525
COLEC11	612502
CYP26B1	605207
EFNA4	601380
EFNB1	300035
ERF	611888
ESCO2	609353
FBN1	134797
FGF9	600921
FGFR1	136350
FGFR2	176943
FGFR3	134934
FREM1	608944
GLI3	165240
GPC3	300037
IFT122	606045
IFT140	614620
IFT43	614068
IGF1R	147370
IL11RA	600939
KAT6A	601408
KAT6B	605880
MASP1	600521
MEGF8	604267
MSX2	123101
NFIA	600727
ORC1	601902
ORC4	603056
ORC6	607213
P4HB	176790
PHEX	300550
POR	124015
PPP3CA	114105
RAB23	606144
RECQL4	603780
RSPRY1	616585
RUNX2	600211
SCARF2	613619
SEC24D	607186
SIX2	604994
SKI	164780

Gene	OMIM ID
SLC25A24	608744
SMAD2	601366
SMAD3	603109
SMAD6	602931
SOX6	607257
SPECC1L	614140
STAT3	102582
TCF12	600480
TCOF1	606847
TGFB2	190220
TGFB3	190230
TGFBR1	190181
TGFBR2	190182
TMCO1	213980
TWIST1	601622
WDR19	608151
WDR35	613602
ZEB2	605802
ZIC1	600470
