# Supplementary figures and images for: TBX3 and EFNA4 Variant in a Family with Ulnar-Mammary Syndrome and Sagittal Craniosynostosis
Source: Genes (Basel). 2022 Sep 14;13(9):1649. doi: 10.3390/genes13091649 (PMC9498434; doi:10.3390/genes13091649)

Wild-type

*TBX3*

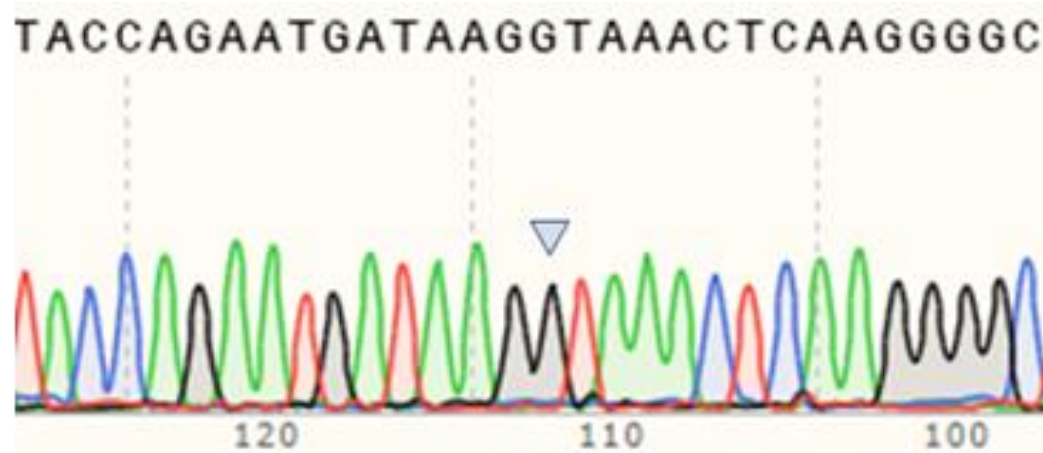

*TBX3* c.804+1 G>A variant

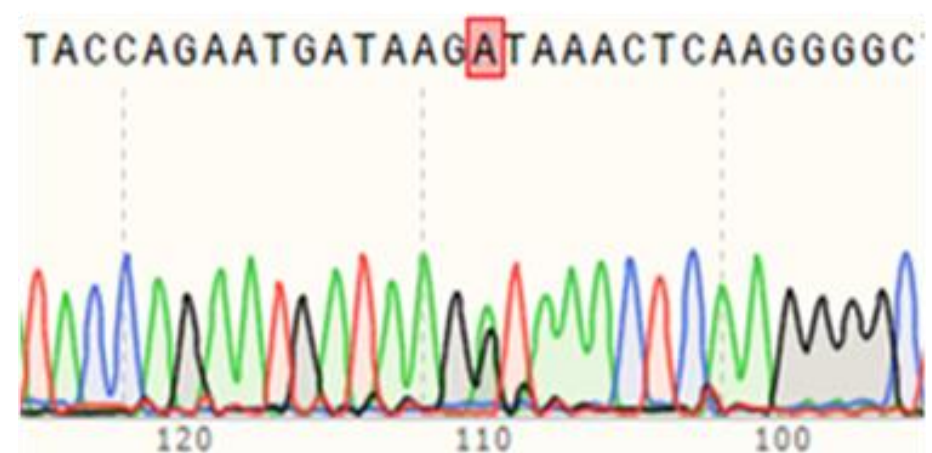

Wild-type

*EFNA4*

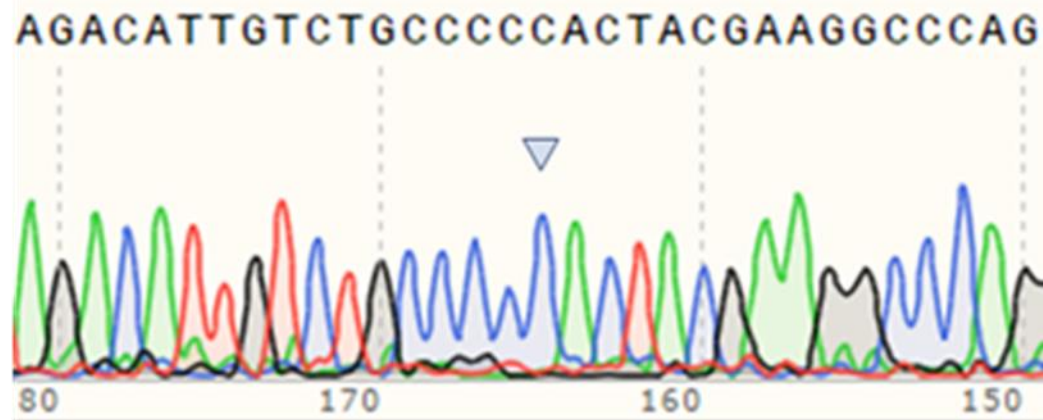

*EFNA4* c.178 C>T variant

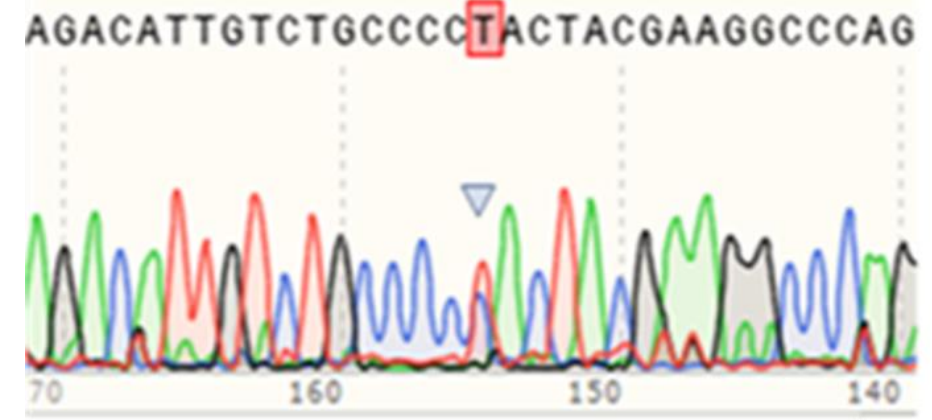

Supplement: Supplementary file 1 [file genes-13-01649-s001.zip › Supplementary Figure S1_Chromatogram.pdf]

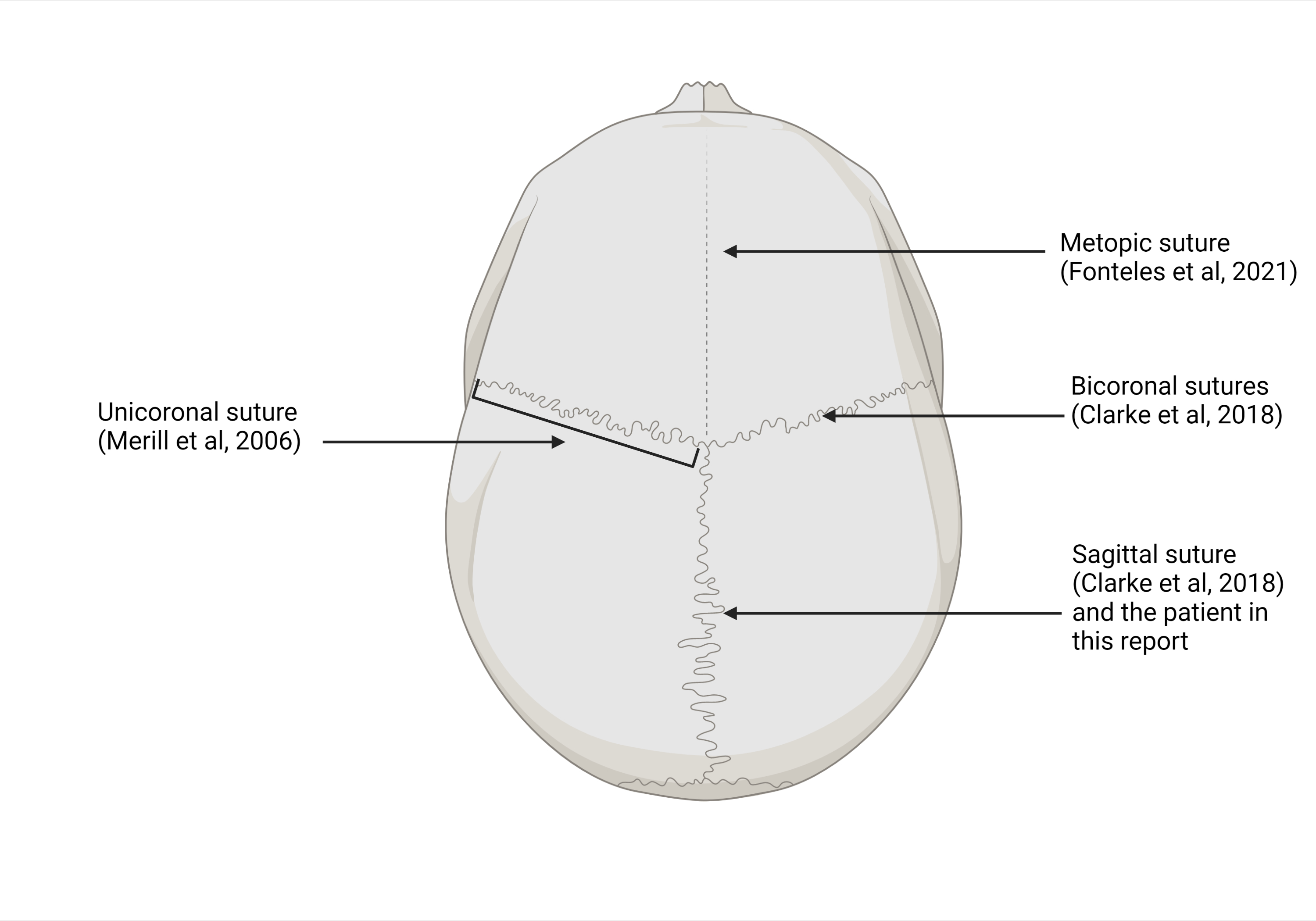

Supplement: Supplementary file 1 [file genes-13-01649-s001.zip › Supplementary Figure S2_EFNA4 c.178C_T reported craniosynostosis (2).jpg]
